# The Deformities of the Fingers and Toes

**Published:** 1898-06

**Authors:** 


**The Deformities of the Fingers and Toes.** By WILLIAM ANDERSON. Pp. viii., 150. London: J. & A. Churchill. 1897.

The groundwork of this book was a course of lectures delivered by Mr. Anderson, at the Royal College of Surgeons, in 1891. It is a valuable contribution to the surgery of deformities.

The important subject of Dupuytren's contraction of the palmar fascia is fully considered, and the various forms of contraction of the fingers described. The very common affection of the great toe known as Hallux valgus is of course referred to, and an interesting account is given of Hallux flexus, a disease only recognised during the last ten years.

The anatomical knowledge of the writer increases the value of his opinion on the pathology and treatment of these deformities, and this little work may be read with advantage by all who have to treat deformities of the fingers and toes. It is clearly written and well illustrated, but we do not like to see even a book of this character without an index.

